# The use of double abdominal braces in knee replacement

**DOI:** 10.1308/003588412X13373405386015i

**Published:** 2012-09

**Authors:** T Woodacre, L Wilkinson, T Ball, RJ Kincaid

**Affiliations:** ^1^South Devon Healthcare NHS Foundation Trust,UK; ^2^Royal Cornwall Hospitals NHS Trust,UK

## BACKGROUND

The correct cementing technique for knee replacement involves maintaining the knee in extension for a protracted period of time to compress the cement effectively during polymerisation.^1,2^ Wound closure can be completed in flexion or extension with no difference in functional outcome^3^ but is often also completed with the leg maintained in extension due to decreased tissue tension. With the increasing average mass of patients, holding a leg in extension can often involve significant energy expenditure. We report an effortless, efficient technique for holding the knee in extension for an indefinite period without the need for surgeon or assistant exertion.

## TECHNIQUE

**Figure 1:**
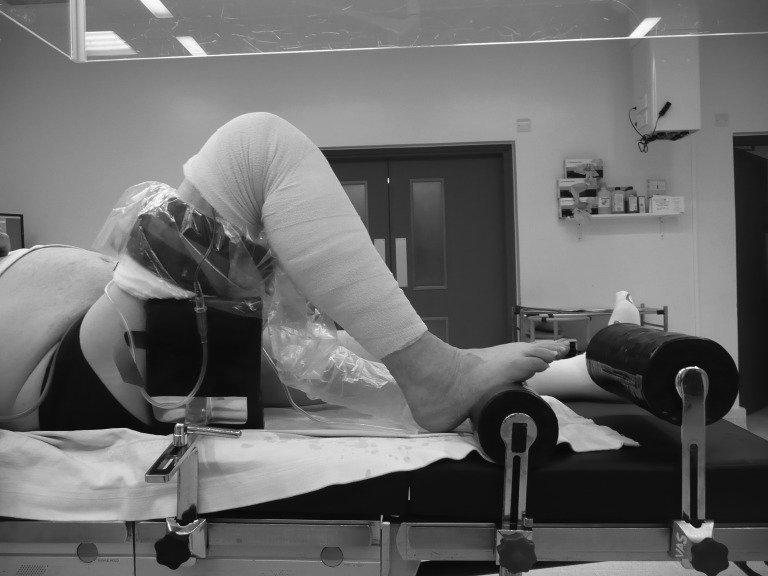
Default position of a knee during total knee replacement with two braces present

**Figure 2:**
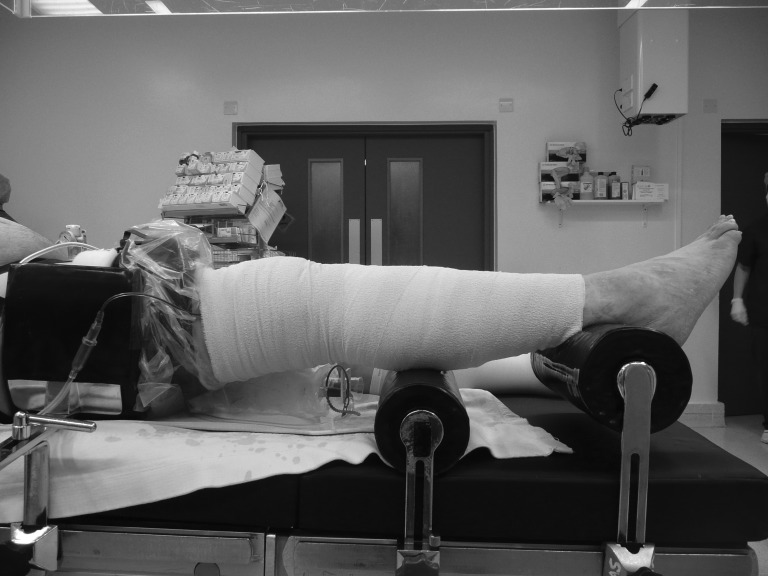
Use of a second brace to maintain a knee in extension

An abdominal brace is used routinely during knee replacement as a foot support to maintain the knee in flexion. Placing a second, larger abdominal brace distally offers no negative interference to surgery (Fig 1). However, when the leg is rested across the brace, it pressurises the knee into extension (Fig 2). A smaller brace positioned distally at a greater height can perform the same function.

## DISCUSSION

We have used this technique in every primary knee replacement performed over the last 18 months. It has facilitated an increased ease of procedure, negating both assistant discomfort when operating on large patients and the need for an assistant during closure. It is now used as standard operative practice by the senior author.

## References

[1] Laskin RS, Riegèr MA. The surgical technique for performing a total knee replacement arthroplasty. Orthop Clin North Am 1989; 20: 31–48 2919077

[2] Insall JN. Total Knee Arthroplasty In: Insall JN, ed.Surgery of the Knee. Philadelphia, PA: Elsevier; 1984

[3] Kavarthapu V, Shenava Y, Koka R, DÕArcy JC, Garikipati R. Closure of knee replacement wound in flexion or extension: does it matter? J Bone Joint Surg Br 2004; 86 (Supp III): 278

